# Anticipating undiagnosed asthma in symptomatic adults with normal pre- and post-bronchodilator spirometry: a decision tool for bronchial challenge testing

**DOI:** 10.1186/s12890-023-02806-9

**Published:** 2023-12-09

**Authors:** Sheojung Shin, George Alex Whitmore, Louis-Philippe Boulet, Marie-Ève Boulay, Andréanne Côté, Céline Bergeron, Catherine Lemière, M. Diane Lougheed, Katherine L. Vandemheen, Gonzalo G. Alvarez, Sunita Mulpuru, Shawn D. Aaron

**Affiliations:** 1 The Ottawa Hospital Research Institute, University of Ottawa, Ottawa, Canada; 2 Desautels Faculty of Management, McGill University, Montreal, Canada; 3 Institut universitaire de cardiologie et de pneumologie de Québec-Université Laval, Québec, QC Canada; 4 The Lung Center, Vancouver General Hospital, Vancouver, BC Canada; 5 Hôpital Sacré-Coeur, Montréal, QC Canada; 6 Queen’s University, Kingston, ON Canada

**Keywords:** Airway hyperresponsiveness, Asthma, Bronchial challenge testing, Respiratory symptoms, Spirometry

## Abstract

**Background:**

Some patients with asthma demonstrate normal spirometry and remain undiagnosed without further testing.

**Objective:**

To determine clinical predictors of asthma in symptomatic adults with normal spirometry, and to generate a tool to help clinicians decide who should undergo bronchial challenge testing (BCT).

**Methods:**

Using random-digit dialling and population-based case-finding, we recruited adults from the community with respiratory symptoms and no previous history of diagnosed lung disease. Participants with normal pre- and post-bronchodilator spirometry subsequently underwent BCT. Asthma was diagnosed in those with symptoms and a methacholine provocative concentration (PC_20_) of < 8 mg/ml. Sputum and blood eosinophils, and exhaled nitric oxide were measured. Univariate analyses identified potentially predictive variables, which were then used to construct a multivariable logistic regression model to predict asthma. Model sensitivity, specificity, and area under the receiver operating curve (AUC) were calculated.

**Results:**

Of 132 symptomatic individuals with normal spirometry, 34 (26%) had asthma. Of those ultimately diagnosed with asthma, 33 (97%) answered ‘yes’ to a question asking whether they experienced cough, chest tightness or wheezing provoked by exercise or cold air. Other univariate predictors of asthma included female sex, pre-bronchodilator FEV1 percentage predicted, and percent positive change in FEV1 post bronchodilator. A multivariable model containing these predictive variables yielded an AUC of 0.82 (95% CI: 0.72–0.91), a sensitivity of 82%, and a specificity of 66%. The model was used to construct a nomogram to advise clinicians which patients should be prioritized for BCT.

**Conclusions:**

Four readily available patient characteristics demonstrated a high sensitivity and AUC for predicting undiagnosed asthma in symptomatic adults with normal pre- and post-bronchodilator spirometry. These characteristics can potentially help clinicians to decide which individuals with normal spirometry should be investigated with bronchial challenge testing. However, further prospective validation of our decision tool is required.

**Supplementary Information:**

The online version contains supplementary material available at 10.1186/s12890-023-02806-9.

## Background

Asthma is a chronic respiratory disease estimated to affect 262 million people globally [1]. Asthma is characterized by symptoms of shortness of breath, wheezing, and coughing, often leading to frequent healthcare visits and absenteeism. A missed diagnosis may lead to non-treatment or under-treatment, with potential negative impacts on quality of life (QOL) [2].

Asthma is subject to both under- and over-diagnosis. Socioeconomic status, under-reporting of symptoms, and diagnostic insensitivity of spirometry were factors noted to affect underdiagnosis [3, 4]. While spirometry is the most accessible and frequently used test to diagnose asthma, normal values are not exclusive [5]. Schneider and colleagues examined the diagnostic ability of office spirometry and found up to 80% of symptomatic patients had no abnormalities on testing [6]. Currently, guidelines suggest alternative diagnoses or further testing if reversible airflow limitation is not demonstrated with spirometry [7]. Bronchial challenge testing (BCT) is usually the preferred method to diagnose asthma in this situation, but it is unclear how many symptomatic adults are appropriately referred.

Guidelines suggest BCT be used when there is clinical suspicion, but no airflow obstruction found with spirometry [8, 9]. An optimal diagnostic value of this test is reported to be achieved when pretest probability of asthma is 30–70% [10]. However, when Dales and colleagues asked chest physicians ordering methacholine BCTs to predict airway hyperresponsiveness based on clinical gestalt, they found no association between predictions and actual test results [11]. A study assessing the pattern of BCT practices in Canada found large variations across the provinces [12], suggesting that BCT referral is often subject to the clinician’s judgement.

Recent studies have assessed potential variables associated with BCT results consistent with asthma [13, 14]. These studies were limited to retrospective chart analyses and assessment of demographic differences via univariate analyses. There have been no prospective studies that help to predict BCT results.

The objective of this study was to find predictors of asthma in symptomatic adults with normal pre- and post-bronchodilator spirometry, and to generate a tool to help clinicians prioritize who should undergo BCT.

## Materials and methods

### Study design

This was a prospective, multicenter sub-study of subjects enrolled in the larger Undiagnosed COPD and Asthma Population (UCAP) case-finding study, performed in five Canadian respiratory centers in accordance with Strengthening the Reporting of Observational Studies in Epidemiology guidelines [15]. Full details of the larger case-finding study [16, 17] and this sub-study cohort [18] have been published previously. Approval from each local ethics committee was obtained. All subjects signed written, informed consent.

### Study participants

Participants to this sub-study were recruited from a large case-finding study aiming to identify asthma or COPD in symptomatic Canadian adults without prior history of diagnosed airway disease [17]. Adults ≥18-years-old, who had no history of previously diagnosed lung or airway disease, were recruited in a two-step process. In the first step, landlines and cellphones within a 90-minute radius of each study site from June 2017 to March 2020 were random-digit dialled. A scripted message questioned if anyone in the household was 18 years or older and had respiratory symptoms (shortness of breath, wheezing, increased mucus or sputum, and/or prolonged cough). If the response was affirmative, they received a call back from the local study coordinator, who then consented and screened the symptomatic individual for study entry. Individuals were screened using the Asthma Screening Questionnaire (ASQ) [19] and the COPD Diagnostic Questionnaire (COPD-DQ) [20]. Symptomatic participants who scored ≥6 points on the ASQ or > 19.5 points on the COPD-DQ were invited to the study site.

At the study site, participants provided written consent and underwent pre- and post-bronchodilator spirometry to determine whether they had airflow obstruction or a significant bronchodilator response. We defined airflow obstruction on spirometry as any of the following criteria: 1) pre-bronchodilator forced expiratory volume in 1 second (FEV_1_) ≤80%, or 2) pre-bronchodilator FEV_1_/forced vital capacity (FVC) ≤0.70 or ≤ LLN. We defined a significant bronchodilator response as FEV_1_ response to 400 mcg inhaled salbutamol ≥12% and ≥ 200 mL. Subjects who did not show evidence of airflow obstruction or a significant bronchodilator response were offered participation in the sub-study. Pregnant or lactating women, participants who had experienced a respiratory tract infection in the 4 weeks preceding the study visit, or those with medical contraindications to methacholine were excluded [21]. Participants who had used inhaled corticosteroids, or systemic immune-suppressive or immune-modulatory drug therapy within 3 months prior to study visit, or antibiotics in the 4 weeks preceding study visit were also excluded. Those subjects who met eligibility criteria for the sub-study returned for a second visit where they underwent measurement of FeNO, methacholine BCT, blood testing, and induced sputum collection.

### Outcomes

The primary study outcome was a positive finding of asthma. Based on ERS 2022 guidelines for diagnosis of asthma in adults who are steroid-naïve [5], those subjects with respiratory symptoms who demonstrated a 20% fall in FEV1 produced by a methacholine provocation concentration (PC_20_) < 8 mg/mL were considered in this study to have asthma [21]. The secondary outcome was airway hyperresponsiveness (AHR), defined as a PC_20_ of < 16 mg/mL [22].

### Data collection

Sociodemographic data, occupational history, comorbid medical history, smoking history, data on healthcare utilization, work/school absenteeism, medication use, family history of asthma, and atopy were collected from all participants. Validated questionnaires were administered to assess symptoms and QOL scores, including: the COPD Assessment Test (CAT) [23], and the St. George’s Respiratory Questionnaire (SGRQ) [24]. For this sub-study, all participants also completed the Asthma Control Questionnaire (ACQ) [25], the Leicester Cough Questionnaire (LCQ) [26], and a questionnaire adapted from the European Community Respiratory Health Survey (ECRHS III) [18, 27]. Blood tests were performed to assess white blood cell count and differential, particularly for eosinophils, as well as total IgE. Methods based on the work of Pizzichini and colleagues were used to perform induced sputum analysis [28]. Slides were sent to one of the participating sites and sputum differential cell counts were performed by an experienced lab technician blinded to the clinical characteristics of participants.

Allergen testing was performed with skin prick tests utilizing a battery of common aeroallergens, with normal saline and histamine as negative and positive controls, respectively. Atopy was defined as a skin wheal diameter > 3 mm to any allergen. In accordance with American Thoracic Society (ATS) guidelines [29], Fractional exhaled Nitric Oxide (FeNO) measurements were made using a NiOX Mino handheld analyzer (Circassia, Morrisville, NC, USA) before any airflow measurements were performed.

Pre- and post-bronchodilator spirometry was performed according to ATS standards [30] by certified study personnel. The FEV1 predicted values and percentage predicted values were calculated using the Global Initiative (GLI-2012) lung function calculator [31]. Methacholine BCT was performed using the tidal breathing method [9, 22].

### Statistical analysis

Baseline demographic characteristics of participants were summarized using frequency distributions for categorical variables and arithmetic means with standard deviations for quantitative variables. Univariate statistical comparisons were made between subjects with asthma and those without. Chi-square tests were utilized to evaluate the association of the asthma outcome with categorical variables. Logistic regression was used to evaluate the association with quantitative variables. The same analyses were performed for comparisons of AHR. Differences were considered significant and retained for further analysis if univariate tests produced *P*-values < 0.05. Univariate statistical comparisons as well as judgments about clinical relevance were used to select a candidate pool of potentially important decision variables. Goodness of fit for logistic regression models was tested using the Hosmer-Lemeshow test. Receiver Operating Characteristic (ROC) curve analyses, including Area Under the Curve (AUC), were conducted to evaluate, compare, and select a final decision model. Several risk cutoffs for the final decision model were selected to compare model performance in terms of sensitivities and specificities. A simple nomogram was constructed to aid a clinician contemplating referral of a patient for BCT. STATA version 17.0 (StataCorp, College Station, TX, USA), was used to complete all statistical analyses.

### The strategy for risk factor selection

We chose a strategy for selecting risk factors for the decision tool that relies heavily on clinical judgment and real-world clinical tests available to practicing clinicians. We decided a priori to limit factor selection to variables readily available in a physician’s practice including: patients’ sociodemographic variables, medical history, medications, respiratory symptom and QOL questionnaires, pre- and post-BD spirometry, with consideration of blood eosinophils and FeNO tests if they proved to be highly important. Clinical considerations primarily guided variable selection, with supplementation by statistical techniques to take variable correlations into account.

## Results

### Study population

**Fig. 1:**
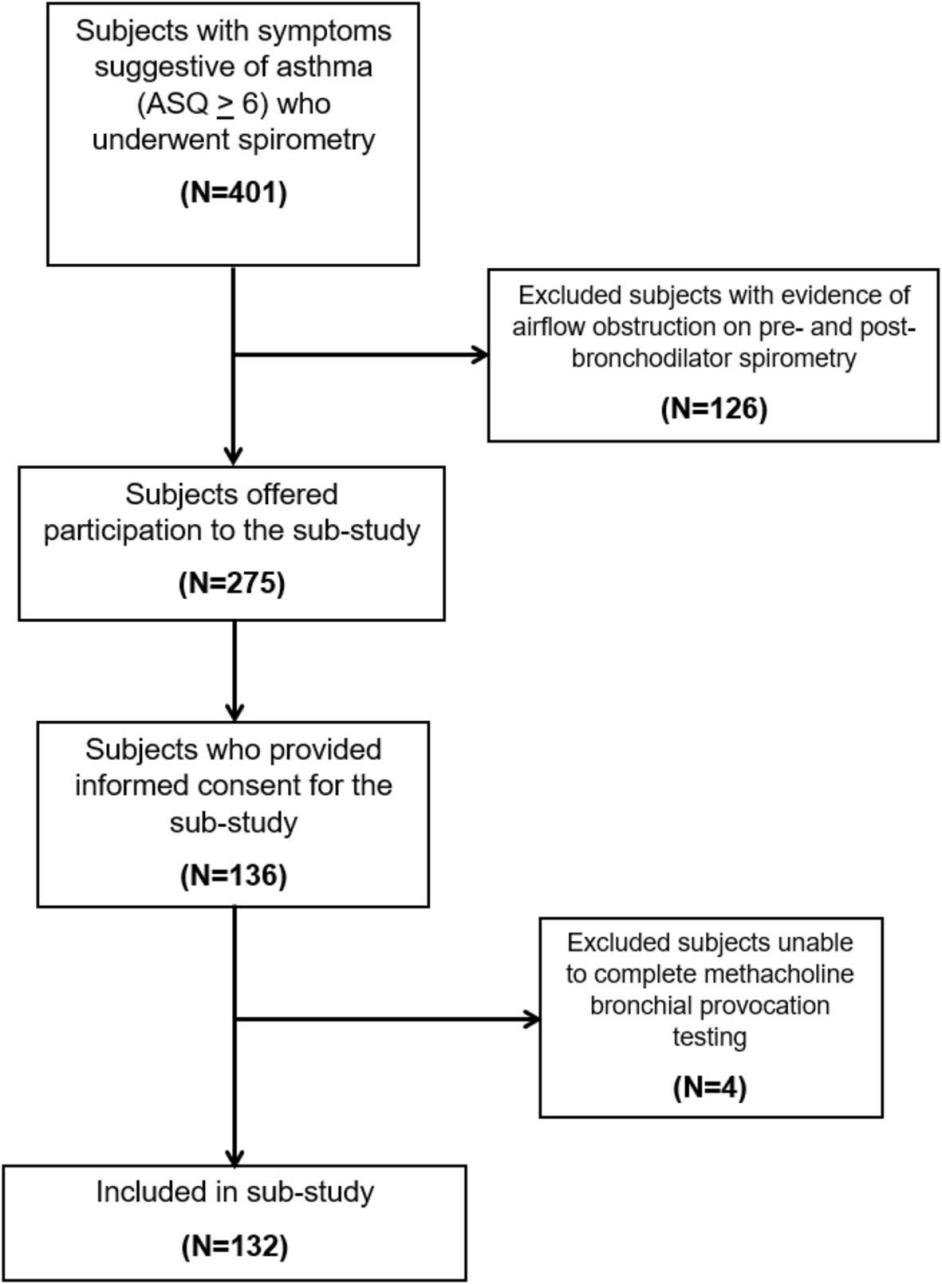
Flow of study participants

The flow of study participants is exhibited in Fig. 1. A total of 275 potentially eligible subjects from the UCAP Study were invited to participate in this sub-study, and 136 provided informed consent for enrolment. Of the 136 consented participants, 4 were excluded because they could not complete BCT. Thus, 132 subjects were included in the analyses.

### Factors associated with asthma

Among the 132 study subjects, 34 (26%; 95% CI 19–34%) had asthma, defined by PC_20_ of < 8 mg/mL. Baseline subject characteristics are exhibited in Table 1, classified by the BCT outcome. A higher proportion of subjects with asthma were female compared to those who tested negative for asthma: 74% (95% CI 56–87%) versus 44% (95% CI 34–54%), *P* = 0.003. There were no significant differences between the two groups when characteristics of age, BMI, smoking history, eczema, atopy, and mean number of allergens were compared. A higher proportion of subjects with asthma had previously used salbutamol for symptoms: 29% (95% CI 15–47%) versus 8% (95% CI 4–15%), *P* = 0.002.

**Table 1:** Subject characteristics

	Subjects without asthma, PC_20_ > 8 mg/mL (*N* = 98)	Subjects with asthma, PC_20_ < 8 mg/mL (*N* = 34)	*P* value
**Age (SD)**	58.1 (14.1)	55.2 (14.7)	0.313
**Female**	43 (43.9%)	25 (73.5%)	**0.003**
**BMI (SD)**	29.9 (5.8)	30.5 (7.1)	0.666
**History of Smoking**	–	–	0.415^a^
*Current smoker (%)*	7 (7.2%)	5 (14.7%)	
*Past smoker (%)*	41(42.3%)	14 (41.2%)	
*Never smoker (%)*	49 (50.5%)	15 (44.1%)	
**History of Eczema**	15 (15.3%)	6 (17.6%)	0.748
**History of Atopy**	45 (52.3%)	19 (59.4%)	0.494
**Number of allergic reactions to allergy skin test**^**b**^ ***(min 0, max 24)*** **(SD)**	4.8 (5.2)	7.0 (6.2)	0.161
Prior use of salbutamol	8 (8.1%)	10 (29.4%)	**0.002**
**Pre bronchodilator FEV1% predicted (SD)**	103.6 (13.2)	96.9 (7.7)	**0.007**
**FEV1% response to bronchodilator (traditional calculation)**^**c**^ **(SD)**	1.9 (4.9)	5.1 (6.0)	**0.004**
**FEV1% response to bronchodilator (new ERS calculation)**^**d**^ **(SD)**	2.1 (3.6)	3.9 (3.2)	**0.018**
**Pre bronchodilator FVC % predicted (SD)**	104.4 (13.5)	99.6 (7.6)	**0.055**
**FVC % response to bronchodilator (traditional calculation)**^**c**^ **(SD)**	−1.0 (3.1)	0.4 (3.3)	**0.027**
**FVC % response to bronchodilator (new ERS calculation)**^**d**^ **(SD)**	−1.1 (3.3)	0.4 (3.4)	**0.033**
**Pre bronchodilator FEV1/FVC**	78.1 (4.0)	77.4 (4.6)	0.403
**Post bronchodilator FEV1/FVC**	80.6 (4.2)	80.3 (4.9)	0.722
**Blood Absolute Neutrophil Count (SD)**	4201 (1322)	3854 (1235)	0.184
**Blood Neutrophil percentage (SD)**	60.3 (7.9)	57.0 (9.0)	**0.044**
**Blood Absolute Eosinophil Count (SD)**	171 (132)	200 (145)	0.290
**Blood Eosinophil percentage (SD)**	2.6 (1.9)	3.0 (2.3)	0.255
**Sputum neutrophil %**^**e**^ **(SD)**	52.5 (25.8)	34.7 (23.9)	**0.010**
**Sputum eosinophil %**^**e**^ **(SD)**	1.6 (2.3)	3.3 (5.1)	0.092
**FeNO (SD)**	21.3 (18.4)	21.6 (18.7)	0.929
**Subjects with FeNO > 25 ppb**	21 (23.6%)	9 (27.3%)	0.675
**CAT total score (SD)**	17.3 (6.9)	17.2 (4.8)	0.944
**SGRQ total score (SD)**	37.3 (18.2)	36.0 (14.6)	0.703
**ACQ5 total score (SD)**	0.9 (1.00)	1.0 (0.9)	0.687
**LCQ total score (SD)**	16.8 (3.2)	16.6 (3.1)	0.696
**Answered yes to GAS question: “When you exercise, work hard physically, or when you inhale cold and dry air during the winter, do you ever cough, feel tightness in your chest, have wheezing or whistling or start to be out of breath?”**	73 (74.5%)	33 (97.1%)	**0.004**

^a^*P*-value for a chi-square test of a difference in frequency distributions for all categories of the variable

^b^62 subjects completed allergen skin testing

^c^FEV1% response to bronchodilator (traditional calculation) = (Post-bronchodilator FEV1 – Pre-bronchodilator FEV1)/ Pre-bronchodilator FEV1

^d^FEV1% response to bronchodilator (new ERS calculation) = (Post-bronchodilator FEV1 – Pre-bronchodilator FEV1) / (FEV1 predicted value)

^e^86 subjects were able to produce sputum for testing

Average pre-bronchodilator FEV1% predicted was notably lower in subjects with asthma 97% (95% CI 94–100%) compared to those without 104% (95% CI 101–106%), *P* = 0.007. Participants with asthma demonstrated a greater mean FEV1 response to bronchodilator 5% (95% CI 3–7%) than the no-asthma group 2% (95% CI 1–3%), *P* = 0.004. The European Respiratory Society (ERS) recently recommended assessment of percentage bronchodilator response using post-bronchodilator FEV1 minus pre-bronchodilator FEV1, divided by the FEV1 predicted value determined using the appropriate Global Lung Function Initiative (GLI) spirometry equation [30, 32]. There was a significantly higher bronchodilator response using the ERS calculation in the asthma group compared with the no-asthma group, with means of 4% (95% CI 3–5%) and 2% (95% CI 1–3%), respectively, *P* = 0.018.

While all 132 patients were able to complete blood tests, 122 (92%) completed FeNO testing, and only 86 (65%) were able to provide induced sputum for analysis. FeNO, blood and sputum eosinophils were not significantly higher among individuals with asthma. Although no significant differences were noted for total scores on CAT, SGRQ, ACQ5, and LCQ questionnaires, a significantly higher proportion of asthma subjects answered ‘yes’ to a single Global Asthma Symptom (GAS) question reading “When you exercise, work hard physically, or when you inhale cold and dry air during the winter, do you ever cough, feel tightness in your chest, have wheezing or whistling or start to be out of breath?” In the asthma group, 97% (95% CI 85–100%) answered ‘yes’ to this question, versus 74% (95% CI 65–83%) in the no-asthma group, *P* = 0.004.

### Decision model for bronchial challenge testing

Eight clinically important variables were selected to analyze in a multivariable model. These variables were: female sex, age, pre-bronchodilator FEV_1_% predicted, FEV_1_% response post-bronchodilator (defined by both traditional and ERS calculations), prior salbutamol use, absolute blood eosinophil count, and GAS question response.

Only 26 of 132 subjects answered ‘no’ to the GAS question. Only 1 subject of the 26 (3.8%) was found to have asthma. Given this, we reasoned that a ‘no’ answer to the GAS question was a reliable screen to exclude asthma. We thus confined our multivariable analysis to the 106 subjects who answered ‘yes’ to the GAS question. Estimation of a multivariate logistic model containing the remaining 7 variables revealed that 4 of the variables (FEV1% response post-bronchodilator based on the ERS calculation, prior salbutamol use, age, and absolute blood eosinophil count) could be eliminated because they did not contribute appreciably to model performance (likelihood ratio test *P* = 0.634). There were no missing data for the remaining variables. The ultimate model (Table 2) for subjects answering ‘yes’ to the GAS question contained the following 3 variables; sex, pre-bronchodilator FEV1% predicted, and the percent change in FEV1 post-bronchodilator. All three variables made strong individual contributions to model performance, as confirmed by their small *P*-values. A Hosmer-Lemeshow test of this model using 10 quantile groups confirmed an acceptable fit (*P* = 0.37).

**Table 2:** Multivariable analysis of risk factors associated with Asthma for subjects responding “Yes” to the GAS question ^a^

Variable	Odds Ratio	Two-sided *P*-value	95% Conf. Interval
Female	4.558	0.003	(1.686, 12.322)
Pre bronchodilator FEV1% predicted ^b^	0.940	0.010	(0.896, 0.985)
FEV1% response to bronchodilator ^c^	1.190	0.019	(1.029, 1.376)
Constant	49.023		

^a^Global Asthma Symptoms (GAS) question; “When you exercise, work hard physically, or when you inhale cold and dry air during the winter, do you ever cough, feel tightness in your chest, have wheezing or whistling or start to be out of breath?”

^b^Calculated using GLI reference values

^c^FEV1% response to bronchodilator (traditional calculation) = (Post-bronchodilator FEV1 – Pre-bronchodilator FEV1)/ Pre-bronchodilator FEV1

**Fig. 2:**
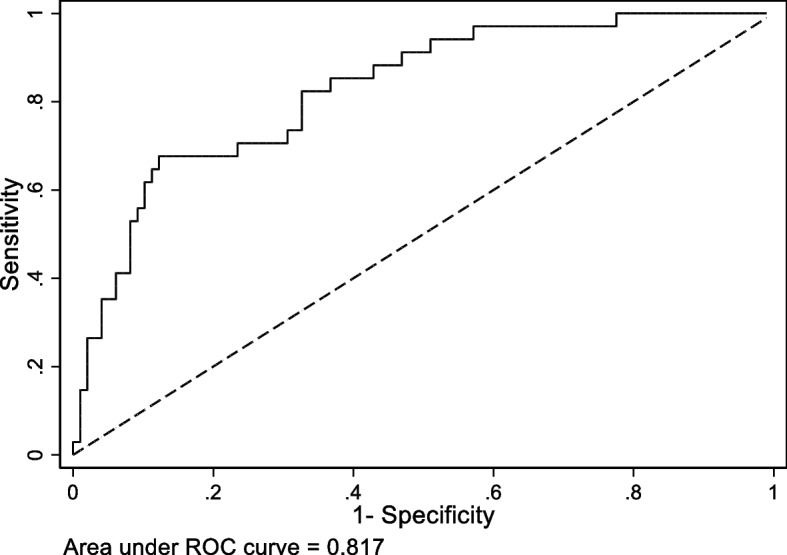
ROC curve for the asthma prediction model

The final analysis was based on results in the two subgroups, the group who answered ‘no’ to the GAS question with 26 subjects and the group who answered ‘yes’ with 106 subjects. When the decision rules for the two subgroups are merged, the model’s ROC curve reflects the composite decision results of the two subgroups and is presented in Fig. 2, where its AUC is shown as 0.82 (95% CI: 0.72–0.91).

To select a risk cutoff for this decision model we considered that a methacholine BCT is costly to the patient and the healthcare system both in direct test costs and indirect lost-time costs of patients undergoing further testing [1–4]. However, given the lifelong morbidity costs associated with undiagnosed/untreated asthma, the cost of a missed diagnosis was deemed to outweigh that of a test used to rule out this diagnosis. We calculated sensitivities and specificities of several risk cutoffs, ranging from 10 to 40% (Table 3). A 20% risk of asthma was selected as having the best balance of sensitivity and specificity. Table 4 presents a 2 × 2 cross-classification of predictions and true disease states for this decision model, incorporating results from both subgroups of subjects. The table shows the 20% risk cutoff has 82% sensitivity and 66% specificity. The table also shows that 46% of subjects are predicted by the model to have asthma, implying that slightly less than half of patients with normal spirometry would be referred for BCT if this model and the 20% risk cutoff were used in clinical practice.

**Table 3:** Sensitivity and specificity of the asthma prediction model at varying risk cut-offs for asthma

Risk cutoff 10%	Risk cutoff 20%	Risk cutoff 30%	Risk cutoff 40%
Sens 94.1% Spec 46.9%	Sens 82.4% Spec 66.3%	Sens 67.6% Spec 77.6%	Sens 64.7% Spec 87.8%

**Table 4:** Classification table of predicted and true disease at a risk cut-off of 20% for asthma

	True Disease: Asthma	True Disease: No Asthma	Total
**Model Prediction: Asthma**	28	33	61
**Model Prediction: No Asthma**	6	65	71
**Total**	34	98	132

Sensitivity = 28/34 = 82%

Specificity = 65/98 = 66%

### Use in clinical practice

**Fig. 3:**
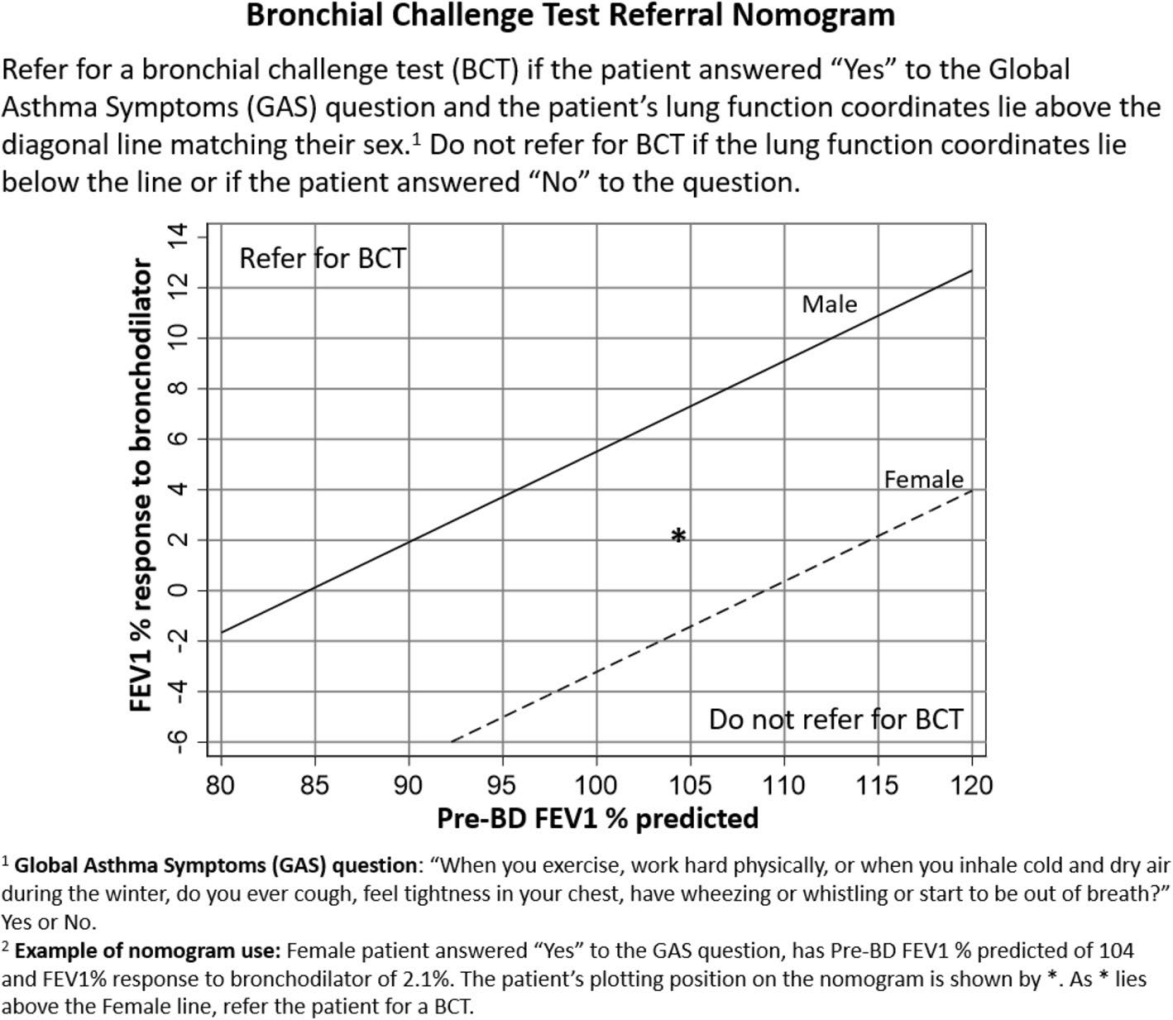
The bronchial challenge test referral nomogram

Figure 3 depicts a nomogram constructed to help determine which symptomatic patients with normal spirometry should be prioritized for BCT. Those patients who answer ‘yes’ to the GAS question and have lung function coordinates that lie above the diagonal line matching their sex would be predicted by the model to have a 20% or higher probability of asthma, and BCT referral should be considered. An example of a study participant is included to illustrate how to use the nomogram.

### Secondary analysis for airway hyperresponsiveness

Among the 132 study subjects, 47 (36%; 95% CI 27–44%) had AHR, defined by PC_20_ of < 16 mg/mL. Baseline characteristics of subjects with AHR and those without AHR are shown in S1 Table. Similar to the subgroups distinguishing asthma diagnosis, female sex, absolute pre-bronchodilator FEV1 values, pre-bronchodilator FEV1% predicted, and FEV1% reversibility post-bronchodilator were predictive of AHR. It was noted that the mean blood absolute eosinophil count was marginally higher in the AHR subgroup 211 cells/mm^3^ (95% CI 160–263) compared to the no AHR subgroup 160 cells/mm^3^ (95% CI 137–183), *P* = 0.049. However, sputum eosinophils and FeNO were not predictive of AHR.

A multivariable logistic model (S2 Table) was constructed in a similar fashion as was done previously for asthma. Unlike the asthma decision model, the GAS question was not dominant in predicting AHR, although its impact is statistically important. Specifically, the AHR multivariable model includes a statistically significant interaction variable for the FEV1 response to bronchodilator and the response to the GAS question. The interaction variable captures how the impact of the FEV1 response variable on AHR risk depends on the response to the GAS question. The strong positive effect is shown by the odds ratio for the interaction variable (OR = 1.367, *P* = 0.003). S3 Table shows the model’s sensitivities and specificities at several risk cutoffs. The 2 × 2 table cross-classifying predictions and true disease states for this model is shown in S4 Table. The table shows the 20% risk cutoff has 89% sensitivity and 42% specificity. The AUC is 0.79 (95% CI: 0.70–0.88), (S1 Figure).

## Discussion

This study explored various clinical and laboratory characteristics of subjects in a population with unexplained respiratory symptoms to help recognize those who may have asthma even when their spirometry results are normal. In this population, 26% had a BCT result in keeping with asthma. We found that a single question exploring respiratory symptoms associated with exertion and cold weather was highly predictive in ruling out potential asthma. When response to this one question was combined with three potential risk factors (sex, pre-bronchodilator FEV1% predicted, and FEV1% response to bronchodilator), a resultant multivariable decision model had high sensitivity for predicting asthma. Use of this simple decision tool can identify patients with normal spirometry whose probability of asthma is ≥20%. Our suggestion is to prioritize these patients for further investigations, including subsequent BCT. Because we are considering adults with significant respiratory symptoms, all patients merit further investigation even if they are not chosen for BCT or their test outcome is negative.

Previous studies have shown that spirometry alone is relatively insensitive to confirm a diagnosis of asthma [3–6]. Louis and colleagues determined in their retrospective study comparing bronchodilator reversibility with methacholine bronchoprovocation testing that while baseline airway obstruction may be predictive of reversibility, it was not predictive of airway hyperresponsiveness [33]. As Busse highlights in his discussion regarding diagnostic approach to asthma, normal baseline lung function does not rule out the diagnosis of asthma, and further testing such as with bronchoprovocation may provide useful clinical information [34]. Despite recommendations for further testing in symptomatic individuals with normal spirometry [5, 35], there is a lack of data regarding which individuals should undergo BCT when spirometry is normal. Our study helps clarify which symptomatic individuals are most likely to test positive for asthma using BCT.

Several recent studies have assessed potential predictors of asthma in symptomatic individuals with normal spirometry. Nickels performed a retrospective chart review of 1322 adults with non-obstructive spirometry results to assess if FeNO testing is predictive of a positive methacholine BCT, which did not reveal any statistically significant findings [36]. Chevrier conducted a retrospective chart review of 1126 subjects with normal spirometry results who underwent methacholine BCT [14]. The authors identified younger age, female sex, body mass index (BMI) > 40, and symptoms of wheezing as factors associated with an abnormal BCT of PC_20_ < 16 mg/mL. Peled and colleagues identified in their retrospective analysis that all baseline spirometry values were significantly lower in the group with airway hyperresponsiveness and highlighted the potential role of the forced mid-expiratory flow rate (FEF_50%_) of predicting AHR [37]. Selvanathan and colleagues, in a large cohort study, assessed the proportion of adults with a positive BCT among those with self-reported physician-diagnosed asthma and a negative bronchodilator response. They found that 43% had a positive BCT. They describe baseline airflow limitation as being predictive of a positive BCT and that the negative predictive value of spirometry with bronchodilator response for ruling out asthma is low [38].

This is the first prospective study to determine useful clinical predictors of asthma in symptomatic subjects with non-obstructive spirometry results. These subjects were a representative sample of individuals selected randomly from the population who reported no prior asthma or airway disease history. Our aim was to provide a decision-making aid for the clinician regarding BCT referral to avoid possible detrimental delays in diagnoses and treatment, as there is evidence that individuals may incur loss in pulmonary function with untreated mild asthma over time [39, 40]. The variability of BCT referral across regions [12] suggests that clinicians may benefit from a standard tool. This tool recognizes that a BCT may be costly [12, 41], but a misdiagnosis of asthma may produce higher costs for the patient and healthcare system [42, 43]. A standard tool may reduce inappropriate referrals, encourage more timely and accurate diagnoses, and in turn potentially diminish costs.

The four factors in our model (sex, FEV_1_% predicted, FEV_1_% response to bronchodilator, and self-reported respiratory symptoms associated with exertion and/or cold weather) are readily accessible in a primary care practice that has access to spirometry testing. These factors are universal and are well known to be associated with asthma widely. Asthma is more prevalent in female adults than male adults [44, 45], and FEV1% predicted, FEV1% response to bronchodilator, and symptoms associated with exertion and/or cold weather are universally utilized for asthma diagnosis and monitoring [32, 46–48]. Our decision tool is easy to utilize in various clinical settings, which could reduce delays in testing and diagnosis. Although FeNO, blood and sputum eosinophils were assessed, these laboratory-based variables were not significant discriminators of asthma in our analyses.

This study identified female sex as a strong predictor for asthma. It is well known that male children are more likely to have asthma than female children, however the opposite is true for adults [41, 42]. Although the pathophysiology is not completely understood, there are many studies characterizing genomic differences and the physiologic hormonal milieu that may impact bronchial response [49, 50]. Of note, we found in our study that FVC and height were comparatively lower in subjects who had asthma compared to those who did not. Physiologically, FVC, height, and female sex are strongly correlated as females have smaller lung volumes than men on average. Given that the BCT measures the methacholine concentration required to cause a 20% fall in FEV_1_ compared to baseline, the question arose as to whether women are more likely to test positive for asthma because of a smaller volume change needed to achieve a 20% decline. This question has been posed and evaluated in multiple studies previously [51]. Leynaert and colleagues tested this hypothesis with multiple analyses adjusting for lung function parameters and concluded that the higher proportion of AHR in women could not be accounted for by their lung volumes alone [52].

Our study has the following limitations. Although sputum variables may be clinically relevant, only 86 of the 132 participants were able to produce induced sputum samples. This variable was not significant in the univariate analysis and was not included in the multivariable model. Our decision model is developed for methacholine challenge testing and therefore may not be reliable for other methods of bronchial provocation. Because of constraints imposed on recruitment and bronchial provocation testing by the COVID19 pandemic our study was halted in March 2020, and our sample size of 132 individuals was not large enough for both model derivation and validation. A further independent study is required to validate the model we have developed. Finally, our recruitment strategy relied on patient-reported symptoms. Individuals with asthma who under-reported or minimized their respiratory symptoms would not have been diagnosed using our methodology.

## Conclusion

This study demonstrated that in a population of subjects with respiratory symptoms suggestive of asthma but with normal pre- and post-bronchodilator spirometry, 26% had asthma when they proceeded to BCT. Predictors of asthma included having respiratory symptoms associated with exertion and/or cold weather, female sex, relatively lower pre-bronchodilator FEV1% predicted, and greater FEV1 response to bronchodilator. A decision model generated from these variables had a high sensitivity and AUC. Our study may help clinicians recognize which subjects with respiratory symptoms who have normal spirometry should be referred for further investigation with BCT.

### Supplementary Information

**Additional file 1: S1 Table.** Subject Characteristics. **S2 Table.** Multivariate analysis of risk factors associated with airway hyperresponsiveness. **S3 Table.** Associated sensitivity and specificity at varying risk cutoffs for airway hyperresponsiveness. **S4 Table.** Classification table of predicted and true disease at risk cutoff of 20% for airway hyperresponsiveness. **S1 Figure.** ROC curve for the prediction model for airway hyperresponsiveness.

## Data Availability

All data generated or analysed during this study are included in this published article and its supplementary information files.
