# Supplementary material for: Anticipating undiagnosed asthma in symptomatic adults with normal pre- and post-bronchodilator spirometry: a decision tool for bronchial challenge testing
Source: BMC Pulm Med. 2023 Dec 9;23:496. doi: 10.1186/s12890-023-02806-9 (PMC10709915; doi:10.1186/s12890-023-02806-9)
Supplement: Supplementary file 1 — Additional file 1: S1 Table. Subject Characteristics. S2 Table. Multivariate analysis of risk factors associated with airway hyperresponsiveness. S3 Table. Associated sensitivity and specificity at varying risk cutoffs for airway hyperresponsiveness. S4 Table. Classification table of predicted and true disease at risk cutoff of 20% for airway hyperresponsiveness. S1 Figure. ROC curve for the prediction model for airway hyperresponsiveness. [file 12890_2023_2806_MOESM1_ESM.docx]

**SUPPORTING INFORMATION**

**S1 Table.** Subject Characteristics

|  | Subjects with no airway hyperresponsiveness, PC_20_ >16mg/mL  (N=85) | Subjects with airway hyperresponsiveness, PC_20_ <16mg/mL  (N=47) | *P* value |
| --- | --- | --- | --- |
| Age (SD) | 58.1 (14.3) | 55.9 (14.2) | 0.388 |
| Female | 35 (41.2%) | 33 (70.2%) | **0.001** |
| BMI (SD) | 29.5 (5.7) | 31.1 (6.8) | 0.138 |
| History of Smoking | - | - | 0.444^a^ |
| *Current smoker (%)* | 6 (7.1%) | 6 (13.0%) |  |
| *Past smoker (%)* | 35(41.1%) | 20 (43.5%) |  |
| *Never smoker (%)* | 44 (51.8%) | 20 (43.5%) |  |
| History of Eczema | 15 (17.6%) | 6 (12.8%) | 0.463 |
| History of Atopy | 37 (50.0%) | 27 (61.4%) | 0.231 |
| Number of allergic reactions to allergy skin test ^b^ *(min 0, max 24)* (SD) | 5.0 (5.5) | 6.0 (5.6) | 0.492 |
| Prior use of salbutamol | 8 (9.4%) | 10 (21.3%) | 0.057 |
| Pre bronchodilator FEV1 % predicted (SD) | 103.8 (13.8) | 98.5 (8.3) | **0.020** |
| FEV1 % response to bronchodilator (traditional calculation)^c^ (SD) | 2.0 (3.5) | 4.1 (7.5) | **0.047** |
| FEV1 % response to bronchodilator (new ERS calculation)^d^ (SD) | 1.9 (3.6) | 3.8 (3.2) | **0.007** |
| Pre bronchodilator FVC % predicted (SD) | 104.4 (13.7) | 101.0 (9.3) | 0.146 |
| FVC % response to bronchodilator (traditional calculation)^c^ (SD) | -1.0 (3.2) | 0.0 (3.2) | 0.082 |
| FVC % response to bronchodilator (new ERS calculation)^d^ (SD) | -1.1 (3.4) | -0.1 (3.2) | 0.103 |
| Pre bronchodilator FEV1/FVC | 78.2 (4.0) | 77.5 (4.5) | 0.394 |
| Post bronchodilator FEV1/FVC | 80.5 (4.1) | 80.5 (4.8) | 0.971 |
| Blood Absolute Neutrophil Count (SD) | 4,168 (1,381) | 4,005 (1,159) | 0.493 |
| Blood Neutrophil percentage (SD) | 60.3 (8.2) | 57.9 (8.3) | 0.123 |
| Blood Absolute Eosinophil Count (SD) | 160.1 (107.6) | 211.5 (172.8) | **0.049** |
| Blood Eosinophil percentage (SD) | 2.5 (1.7) | 3.1 (2.4) | 0.093 |
| Sputum neutrophil %^e^ (SD) | 54.3 (25.8) | 36.4 (23.8) | **0.004** |
| Sputum eosinophil %^e^ (SD) | 1.7 (2.4) | 2.7 (4.4) | 0.217 |
| FeNO (SD) | 19.9 (12.8) | 23.8 (25.1) | 0.275 |
| Subjects with FeNO >25 ppb | 17 (22.4%) | 13 (28.3%) | 0.464 |
| CAT total score (SD) | 16.9 (6.6) | 17.8 (6.0) | 0.447 |
| SGRQ total score (SD) | 36.3 (17.8) | 38.3 (16.4) | 0.513 |
| ACQ5 total score (SD) | 0.9 (0.9) | 1.1 (0.9) | 0.314 |
| LCQ total score (SD) | 17.1 (3.1) | 16.1 (3.2) | 0.099 |
| Answered yes to the GAS question: “When you exercise, work hard physically, or when you inhale cold and dry air during the winter, do you ever cough, feel tightness in your chest, have wheezing or whistling or start to be out of breath?” | 66 (77.6%) | 40 (85.1%) | 0.302 |

1. *P*-value for a chi-square test of a difference in frequency distributions for all categories of the variable
2. 62 subjects completed allergen skin testing
3. FEV1 % response to bronchodilator (traditional calculation) = (Post-bronchodilator FEV1 – Pre-bronchodilator FEV1)/ Pre-bronchodilator FEV1
4. FEV1 % response to bronchodilator (new ERS calculation) = (Post-bronchodilator FEV1 – Pre-bronchodilator FEV1) / (FEV1 predicted value)
5. 86 subjects were able to produce sputum for testing

**S2 Table.** Multivariate analysis of risk factors associated with airway hyperresponsiveness

| **Variable** | **Odds Ratio** | **Two-sided *P*-value** | **95% Conf. Interval** |
| --- | --- | --- | --- |
| Female | 3.938 | 0.001 | (1.691, 9.172) |
| GAS question^a^ | 0.639 | 0.456 | (0.197, 2.076) |
| Pre bronchodilator FEV1 % predicted^b^ | 0.957 | 0.022 | (0.921, 0.994) |
| FEV1 % response to bronchodilator^c^ | 0.925 | 0.269 | (0.806, 1.062) |
| FEV1 % response to bronchodilator x GAS^d^ | 1.367 | 0.003 | (1.115, 1.676) |
| Constant |  |  |  |

1. Global Asthma Symptoms (GAS) question; “When you exercise, work hard physically, or when you inhale cold and dry air during the winter, do you ever cough, feel tightness in your chest, have wheezing or whistling or start to be out of breath?”
2. Calculated using GLI reference values
3. FEV1 % response to bronchodilator (traditional calculation) = (Post-bronchodilator FEV1 – Pre-bronchodilator FEV1)/ Pre-bronchodilator FEV1
4. FEV1 % response to bronchodilator x GAS interaction: Equals FEV1 % response to bronchodilator if GAS response is “Yes”; equals 0 if response is “No”

**S3 Table.** Associated sensitivity and specificity at varying risk cutoffs for airway hyperresponsiveness

| **Risk cutoff 10%** | **Risk cutoff 20%** | **Risk cutoff 30%** | **Risk cutoff 40%** |
| --- | --- | --- | --- |
| Sens 100.0%  Spec 17.6% | Sens 89.4%  Spec 42.4% | Sens 76.6%  Spec 61.2% | Sens 66.0%  Spec 76.5% |

**S4 Table.** Classification table of predicted and true disease at risk cutoff of 20% for airway hyperresponsiveness

|  | **PC20 < 16 mg/mL** | **PC20 ≥ 16 mg/mL** | **Total** |
| --- | --- | --- | --- |
| **Model Prediction: Bronchial hyper-responsiveness** | 42 | 49 | 91 |
| **Model Prediction: No bronchial hyper-responsiveness** | 5 | 36 | 41 |
| **Total** | 47 | 85 | 132 |

Sensitivity = 42/47 = 89%

Specificity = 36/85 = 42%

**S1 Figure.** ROC curve for the prediction model for airway hyperresponsiveness
